# CD8^+^ T Cell Senescence: Lights and Shadows in Viral Infections, Autoimmune Disorders and Cancer

**DOI:** 10.3390/ijms23063374

**Published:** 2022-03-21

**Authors:** Valentina Tedeschi, Giorgia Paldino, Martina Kunkl, Marino Paroli, Rosa Sorrentino, Loretta Tuosto, Maria Teresa Fiorillo

**Affiliations:** 1 Department of Biology and Biotechnology ‘Charles Darwin’, Sapienza University, 00185 Rome, Italy; giorgia.paldino@uniroma1.it (G.P.); martina.kunkl@uniroma1.it (M.K.); rosa.sorrentino@uniroma1.it (R.S.); loretta.tuosto@uniroma1.it (L.T.); mariateresa.fiorillo@uniroma1.it (M.T.F.); 2 Laboratory Affiliated to Istituto Pasteur Italia-Fondazione Cenci Bolognetti, Sapienza University, 00185 Rome, Italy; 3 Department of Clinical, Internistic, Anesthesiological and Cardiovascular Sciences, Sapienza University, 00185 Rome, Italy; marino.paroli@uniroma1.it

**Keywords:** immune-senescence, inflammaging, CD28^−^ CD57^+^ CD8^+^ T cells, HIV, CMV, SARS-CoV-2, infection, autoimmune disease, cancer

## Abstract

CD8^+^ T lymphocytes are a heterogeneous class of cells that play a crucial role in the adaptive immune response against pathogens and cancer. During their lifetime, they acquire cytotoxic functions to ensure the clearance of infected or transformed cells and, in addition, they turn into memory lymphocytes, thus providing a long-term protection. During ageing, the thymic involution causes a reduction of circulating T cells and an enrichment of memory cells, partially explaining the lowering of the response towards novel antigens with implications in vaccine efficacy. Moreover, the persistent stimulation by several antigens throughout life favors the switching of CD8^+^ T cells towards a senescent phenotype contributing to a low-grade inflammation that is a major component of several ageing-related diseases. In genetically predisposed young people, an immunological stress caused by viral infections (e.g., HIV, CMV, SARS-CoV-2), autoimmune disorders or tumor microenvironment (TME) could mimic the ageing status with the consequent acceleration of T cell senescence. This, in turn, exacerbates the inflamed conditions with dramatic effects on the clinical progression of the disease. A better characterization of the phenotype as well as the functions of senescent CD8^+^ T cells can be pivotal to prevent age-related diseases, to improve vaccine strategies and, possibly, immunotherapies in autoimmune diseases and cancer.

## 1. Introduction

As we get older, the immune system undergoes several changes in order to direct the energy into specific pathways and thus guarantee a healthy ageing. The terms ‘immune-ageing’ or ‘immune-senescence’ refer to the reduced ability of each component of the immune system to maintain homeostasis and to react to stress signals during ageing [1]. Thymic involution is one of the main hallmarks of an aged immune system. In fact, from puberty to about the sixth decade of life, the thymus experiences a process of atrophy that results in the reduction of circulating naïve T lymphocytes and in impaired oligoclonal variability [2,3]. In addition, the repeated exposure to stress and pathogens throughout life favors a persistent activation of the immune system with a consequent basal state of inflammation, defined as ‘inflammaging’ [4]. Such basal inflammation may turn into a persistent and chronic inflammation under specific conditions such as autoimmune diseases, chronic viral infections or tumors, thus leading to an accelerated immune-senescence even in young people.

As mentioned above, the decline in the number of circulating T cells during ageing is accompanied by a change in the cell subset composition [5]. Usually, the naïve/memory phenotype of CD8^+^ T cells is determined by means of specific markers, such as C-C motif chemokine receptor (CCR)-7 and CD45RA, which allow the identification of four sub-populations: Naïve (N) (CCR7^+^ CD45RA^+^), Central Memory (CM) (CCR7^+^ CD45RA^−^), Effector Memory (EM) (CCR7^−^ CD45RA^−^) and Terminally differentiated Effector Memory cells which re-express CD45RA (TEMRA) (CCR7^−^ CD45RA^+^) [6]. In particular, CD8^+^ T cells are likely to be more sensitive to both phenotypic and functional variations during ageing with a faster acquisition of a senescent state compared to the CD4^+^ T cell counterpart [7]. As an explanation, Callender et al. suggested a higher mitochondrial dysfunction in TEMRA CD8^+^ T cells, mainly due to a reduced mitochondrial mass. This compromises their metabolic stability, thus impairing the nutrient uptake and making such T cell subset less resistant to the effects of ageing [8]. During ageing, the frequency of memory T cells increases with respect to the naïve counterpart with a progressive downregulation of markers, such as CD27 and CD28, peculiar of the naïve cells [6,9]. Although CD28 is required for an optimal activation of T lymphocytes, its down-modulation in the memory T cell subset does not result in anergy, but rather in an increased cytotoxic activity [10]. In addition, along with the loss of CD28, memory CD8^+^ T cells acquire CD57, a peculiar marker defining highly differentiated/senescent cells, and upregulate lysosomal senescence-associated β-galactosidase (SA-βGal) activity [11]. Albeit CD28^−^ CD57^+^ CD8^+^ T cells are a distinctive trait of an aged immune system, in young adults, this subset could reach around 20–30% of the overall CD8^+^ T cell compartment compared to the 50% found in the elderly [12]. Moreover, a recent study aimed at profiling the ageing immune system by integrated technologies (single-cell RNA sequencing, flow cytometry, mass cytometry, T Cell Receptor (TCR) and B Cell Receptor (BCR) sequencing, cellular indexing of transcriptomes and epitopes) described two major age-associated CD8^+^ T cell subpopulations, namely: CD27^+^ CD28^+^ CD57^−^ expressing the granzyme K (GZMK^+^) and found in the EM and CM cell compartments, and CD27^−^ CD28^−^ CD57^+^ expressing the granzyme B (GZMB^+^) and present in the EM and TEMRA compartments. Interestingly, the GZMK^+^, but not the GZMB^+^, CD8^+^ T cell subset accumulated in healthy elderly subjects and positively correlated with the plasma levels of IL-6, TNF-α and IL-8, which are typical markers of inflammaging [13]. Nevertheless, the presence of terminally differentiated T lymphocytes with senescent features even in non-elderly people can be ascribed to the persistence of several pathogens whose antigens induce repeated cycles of cell proliferation [14].

Once a T lymphocyte becomes a memory/highly differentiated/senescent cell, the lack of CD27 and CD28 leads to the upregulation of p16 and p21, two proteins crucial for cell cycle regulation. These proteins inhibit cyclins and cyclin-dependent kinases involved in the G1 to S phase transition, resulting in the G1 arrest with consequent replicative senescence [15]. Furthermore, the down-modulation of CD27 and CD28 correlates with the loss of human telomerase reverse transcriptase (hTERT), with a consequent decrease of telomerase activity and increase of the telomere frailty [16]. In particular, a previous work by Plunkett and colleagues suggested that, in highly differentiated CD27^−^ CD28^−^ CD8^+^ T cells, the impairment of telomerase activity was not due to a lack of hTERT *per se*, but rather to a decreased phosphorylation of Akt (Ser473) that, in turn, altered the phosphorylation of hTERT [17]. However, CD28^−^ CD8^+^ T cells are still able to proliferate, probably upon activation of other co-stimulatory pathways. Therefore, these senescent cells do not necessarily fail to proliferate but rather they go through limited but functional cycles of replication, especially during a persistent antigen stimulation, as occurs during chronic viral infections [14].

A recent study reported that, during the ageing process, in healthy individuals, the CD27^−^ CD28^−^ CD8^+^ T cells lost TCR signaling activity and acquired the expression of the activating Natural Killer (NK) receptor NKG2D in complex with the NK adaptor molecule DAP12, becoming cytotoxic towards a wide spectrum of cells expressing NKG2D ligands [18]. Very interestingly, this process is promoted by sestrin-2, a stress-responsive protein that can reprogram non-proliferating senescent-like CD8^+^ T cells to NK-like cells with cytotoxic activity through a still unclear mechanism [18]. The reprogramming of these senescent CD8^+^ T cells from a TCR to a NK receptor-mediated functional activity can be crucial for maintaining a long-lasting protection against persistent viruses such as Cytomegalovirus (CMV) and Epstein–Barr Virus (EBV), but also for immune surveillance against transformed cells. Moreover, these senescent CD8^+^ T cells, by acquiring innate-like properties, can eliminate other senescent cells through a NKG2D-dependent killing mechanism, thus contrasting their accumulation in aged tissues and reducing age-related organ dysfunction [18].

Senescent CD8^+^ T cells are not only favored by an inflammatory milieu but may fuel it by producing several pro-inflammatory molecules known as contributors of the Senescence-Associated Secretory Phenotype (SASP) [19,20]. Each senescent cell subset is provided with a specific SASP and, in particular, CD8^+^ T cells mostly secrete proteases, interleukins, chemokines and pro-inflammatory factors rather than growth factors [19]. Moreover, it has been found that, among the markers used to define the senescent CD8^+^ T cell subpopulations, CD57 expression correlates with the production of granzyme A, granzyme B and perforin, thus supporting the highly cytotoxic potential of these senescent cells [21]. Interestingly, senescent CD8^+^ T cells are also good producers of IFN-γ and a positive correlation between the IFN-γ production and age as well as with the frequency of CD28^−^ CD57^+^ CD8^+^ T lymphocytes has been observed [22]. Notably, the cytotoxic potential of senescent CD8^+^ T cells is regulated by two transcription factors, T-bet and Eomes, which are responsible for the effector functions of CD8^+^ T lymphocytes and, in particular, for the production of granzyme B, granzyme H and perforin. Not surprisingly, a higher level of T-bet and Eomes was observed in CD28^−^ CD8^+^ T lymphocytes [23]. In turn, the expression of T-bet and Eomes is controlled by mTOR, a key regulator of cellular metabolism that is crucial for the development of CD8^+^ T cells [24]. Although the inhibition of mTOR seems to ameliorate several age-related diseases in old mice, human senescent CD8^+^ T cells still maintain their effector functions independently from mTOR [25,26,27]. In fact, even though rapamycin, one of the widely used mTOR inhibitors, induced a reduction of IFN-γ production in less differentiated cells, the same effect was not observed in senescent CD8^+^ T cells, suggesting that other mechanisms, rather than mTOR pathway, come into play during senescence [27].

The immune-senescence is a complex biological process during which the immune cells acquire several alterations due to the escape from the classical regulating pathways. Accordingly, gene expression analysis of senescent CD8^+^ T cells demonstrated a downregulation of several genes involved in the regulation of RNA transcription, RNA and DNA metabolism, intracellular transportation, signal transduction pathways and ubiquitin cycle, thus suggesting that the age-related changes negatively impact on DNA and RNA stability with consequences on several biological functions [28]. Moreover, some circumstances, such as inflammation due to viral infections, autoimmune conditions or cancer, could resemble the normal ageing and accelerate the senescence of T cells regardless of the chronological age [29]. In this review, we will focus on the vicious cycle between the processes inducing CD8^+^ T cell senescence and the inflammatory environments.

## 2. The ‘Inflammaging’ of CD8^+^ T Cells during Viral Infections: Focus on HIV, CMV and SARS-CoV-2 Cases

The age-related changes of the immune system are characterized by a decline of the adaptive immunity and a mild hyper-activation of innate immunity that concur to the inflammaging process. These changes are induced by many intrinsic and extrinsic potential factors, including lifelong exposure to pathogens and antigens. In this regard, a major role is played by viruses establishing a persistent infection, such as CMV and Human Immunodeficiency Virus (HIV), that strongly contribute to ageing and immune-senescence acceleration.

In people living with HIV infection (PLWH), an enrichment of a specific memory CD8^+^ T cell subset expressing CD57, a classical senescence marker, and CX3CR1, the receptor for fractalkine, was found to be dependent on coinfection with CMV [30]. Although these cells exhibited a senescent phenotype characterized by the loss of CD28, the acquisition of CD57 and a high expression of T-bet, they were still able to proliferate upon IL-15 stimulation and to maintain effector functions with production of granzyme B and perforins [30]. Notably, during chronic HIV infection, high levels of IL-15 correlate significantly with HIV viremia and the markers of inflammation, thus suggesting that such ‘inflammescent’ subset could be induced by an inflammatory milieu and may contribute to sustain it [31,32]. Hepatitis C Virus (HCV) and Hepatitis B Virus (HBV), besides CMV, are frequently detected in combined antiretroviral therapy (cART) treated PLWH, supporting the notion that a constant antigen exposure may contribute to a dysfunctional immune-senescence [33]. In this regard, Hove-Skovsgaard and colleagues enrolled well-treated PLWH with no detectable HBV or HCV infection to establish whether residual CD8^+^ T cell dysfunctions (increased activation, senescence and apoptosis), resembling those observed during ageing, could be due to the HIV status. Interestingly, they found a higher proportion of activated CD8^+^ T cells (CD38^+^/HLA-DR^+^) with apoptotic features (CD28^−^/CD95^+^) in PLWH compared to healthy subjects [34]. Additionally, it has been shown that HIV persistence under ART is associated with a subset of senescent CD8^+^ T cells (CD27^−^ CD28^−^ CD57^+^) that down-modulates the T-cell immunoglobulin-like receptor CD96 and displays lower proliferative capacity and suboptimal responses to HIV antigens [35]. Indeed, HIV-1 persistence contributes to a premature immune-senescence [36], often exacerbated by CMV coinfection [37,38], and a higher susceptibility to age-associated multi-morbidity [39]. Interestingly, children with perinatally acquired HIV-1 (PHIV) also face a premature T cell senescence that could be limited by an early administration of ART, which prevents viremia explosion [40,41].

CMV could be considered as a leader among the triggering agents of immune-senescence [42]. Throughout life, CMV-related chronic infections lead to an atypical expansion of CMV-specific memory CD8^+^ T cells that retain an active metabolism while expressing a senescent phenotype [42]. In addition, these cells may also upregulate inhibitory receptors, such as Programmed cell Death protein (PD)-1 and Cytotoxic T-Lymphocyte-Associated protein (CTLA)-4, which are often co-expressed by exhausted cells [43]. In this regard, a recent study described two subsets of stem-like CD8^+^ memory T cell progenitors, the CCR7^+^/PD-1^−^/TIGIT^−^ (T cell immunoreceptor with Ig and ITIM domains) T_STEM_, committed to a functional lineage and relatively resistant to exhaustion, and the CCR7^+^/PD-1^+^/TIGIT^+^ T_PEX_ with a dysfunctional and exhaustion-like signature. Interestingly, the T_PEX_ subset is highly enriched among high-avidity CMV-specific or, even more, among EBV-specific CD8^+^ T cell populations [44]. A better characterization of these cell subsets may be useful to identify novel therapeutic intervention to inhibit or reverse T cell exhaustion.

Moreover, as a consequence of the massive clonal proliferation, CMV-specific CD8^+^ T cells, while down-modulating CD28 and acquiring CD57, undergo telomere attrition but maintain the capability to sustain the cytotoxic responses [45,46]. The high frequency of these cells may represent a link between CMV chronic infection and age-related complications even in younger people, due to the immune alterations, which usually occur with ageing [47].

The debate about a possible acceleration of cellular senescence following HIV infection or HIV/CMV coinfection is relevant in the context of the novel coronavirus disease (COVID-19) as well. In this regard, a strong correlation between age and COVID-19 severity is recognized worldwide [48]. The dysregulated immune system of the elderly, characterized by a low-grade inflammatory state with increased levels of IL-6, IL-1RA, TNF-α, IL-1 and C-reactive protein (CRP) in the serum, telomeric alterations, oxidative stress, activation of inflammasome and autophagy pathways, may be responsible for both the impaired defense to the virus and the harmful inflammatory responses, which contribute to the lung tissue injury [49,50]. In particular, such correlation may rely on the uncoordinated adaptive responses of Sars-CoV-2-specific CD4^+^ and CD8^+^ T cells and on the paucity of naïve CD8^+^ and CD4^+^ T cell pool in the elderly, which leads to an unsuccessful response to new viral antigens [51]. In this context, an in vitro study, performed to understand the age-related decline of primary CD8^+^ T cell response in COVID-19 [52], showed that immune-aging, by affecting the percentage of circulating naïve CD8^+^ T cells, weakened the primary response in terms of magnitude, intensity and number of recognized epitopes and thus increasing disease severity [52]. In addition, Nicoli et al. correlated the susceptibility to apoptosis and the suboptimal proliferation to TCR stimulation with higher levels of basal activation and with dysregulated basal lipid metabolism in naïve CD8^+^ T cells from elderly humans [53]. Notably, some lipid-altering drugs have been proved to restore antigen responsiveness of naïve CD8^+^ T cells, thus suggesting new supportive therapeutic approaches to potentiate adaptive immunity against emerging pathogens and targetable tumors.

Intriguingly, a low percentage of naïve CD4^+^ T cells has been found in acute COVID-19 patients, while the paucity of naïve CD8^+^ T cells is correlated with the severity of the disease in all patients [51]. On the other hand, an enrichment of activated CD38^+^ CD8^+^ T cells expressing CD57 and PD-1 has been observed, thus suggesting an increase of senescent and exhausted CD8^+^ T cells in COVID-19 patients [54,55,56]. Moreover, it has been observed that CD8^+^ T cells from COVID-19 patients are able to produce granzyme B, CD107a, IL-17A, IL-2, TNF-α and IFN-γ [54], fueling the cytokine storm occurring in severe cases [57].

On the other hand, in non-elderly patients with a mild COVID-19 disease course, Sars-Cov-2 infection induces the expansion of effector CD8^+^ T cells producing cytotoxic molecules such as granzymes A and B and perforin [58]. Notably, in these patients, PD-1^+^ CD8^+^ T cells exhibit a cytotoxic profile, thus suggesting that they have not acquired a functionally exhausted phenotype. Hence, it can be speculated that PD-1 expression on multifunctional cytotoxic T cells may act as a negative switch for stronger and/or prolonged responses during acute SARS-CoV-2 infection. Contrary to the findings in younger patients, no clear cytotoxic CD8^+^ T cell responses were observed in COVID-19 patients over the 80 years old [58]. This might be explained by the age-dependent decline of circulating CD8^+^ T cell frequencies and by the higher baseline expression levels of granzymes and perforin naturally occurring in healthy older subjects (80–96 years old).

It must be considered that the altered immune response against SARS-CoV-2 could also depend on the telomere shortening of T cells, a feature commonly occurring in older subjects, even though young individuals with an inherent telomere short length could be more predisposed to a severe outcome [59,60,61].

## 3. The Impact of Autoimmunity on the Progression of CD8^+^ T Cells to Senescence

The chronic inflammation observed in several autoimmune diseases may also overlap with the low-grade inflammation found in elderly people [62]. In particular, IL-6, a pro-inflammatory cytokine implicated in the cytokine storm occurring in severe COVID-19, is upregulated in both inflammaging and Rheumatoid Arthritis (RA), a chronic autoimmune rheumatic disease [62,63,64,65,66]. In addition, the cartilage tissue-invasive CD4^+^ T cells from patients with RA showed a significant telomere frailty, a senescence hallmark, due to a low expression of the double-strand-break repair nuclease Meiotic Recombination 11 Homolog A (MRE11A) [67]. Such damage and, in turn, the invasiveness, were reverted by MRE11A over-expression, suggesting that preventing the premature senescence of T cells could be beneficial for the disease treatment [67]. Furthermore, an accumulation of CD28^−^ CD4^+^ and CD28^−^ CD8^+^ T cells has been described in RA patients [68,69] and, notably, therapy with abatacept, a CD80/86-CD28 T cell co-stimulation modulator, was able to reduce these T cell subsets with an improvement of the disease, as measured by DAS28 score and CRP levels [70].

Interestingly, ageing is also accompanied by an increased susceptibility to a broad panel of pathologies, including autoimmune conditions [71,72]. Accordingly, it might be worth investigating whether premature immune-senescence could be primary in an autoimmune disease or secondary to the chronic inflammatory processes. Moreover, several autoimmune disorders are related to certain pathogens that could further challenge the immune system potentiating the activation of the cells with a consequent senescent effect [73].

CMV is also strikingly implicated in autoimmune disorders, albeit the exact mechanisms favoring the break of self-tolerance are still unclear [74]. Likely, CMV may exacerbate the immune-senescence by favoring the expansion of overstimulated T subsets. In particular, the persistence of CMV in subjects affected by RA seems to promote the increase of CX3CR1-expressing CD28^null^ CD4^+^ T cells with altered cytotoxic functions that could reach the synovial fluid and contribute to the tissue damage [75]. In addition, in RA patients, an expansion of CMVpp65-specific CD28^−^ CD8^+^ T cells producing high levels of IFN-γ was found, thus contributing to the overall inflammation [76]. Likewise, in patients with Multiple Sclerosis (MS), the CMV seropositivity has been related to a higher frequency of cytotoxic CD28^null^ CD4^+^ T cells [77] and CMV-restricted CD8^+^ T cells have been found in MS lesions [78]. Similarly, pre-existing CMV infection promoted the expansion of CD28^−^ CD8^+^ T cells during the progression of Type 1 Diabetes (T1D). The increased frequency of these CD28^−^ CD8^+^ T cells in T1D was also related to the death of pancreatic beta cells, thus reinforcing the contribution of such herpesvirus in switching the T compartment towards a senescent profile with consequences on the disease severity in genetically predisposed subjects [79].

Furthermore, CD8^+^ CD57^+^ T cells, highly expressing PD1, have been detected in patients with MS in the inactive disease phase [80]. These cells with an overt senescent/exhausted phenotype were unable to control the replication of EBV that is considered a putative trigger of MS [81,82]. These findings suggest that T cell exhaustion may cause an inefficient EBV immune response during the remission phase, thus favoring virus reactivation in the central nervous system and, consequently, disease exacerbation.

However, during an autoimmune process, the co-presence of a virus is not a *conditio sine qua non* for progression of T cells towards senescence. In fact, autoimmunity *per se* implies an alteration of both innate and adaptive cell compartments with production of pro-inflammatory cytokines that may favor T cell senescence. In turn, such T cells characterized by a specific SASP may worsen the clinical picture by sustaining a dysregulated and constant activation of the immune system. Recently, two papers have reported early immune-senescence in patients with Systemic Lupus Erythematosus (SLE) with dramatic consequences on the disease severity. Indeed, a correlation between senescent T cells, described as CD28^−^ CD57^+^ Killer cell Lectin-like Receptor subfamily G (KLRG)-1^+^ CD8^+^ T cells, and cognitive defects in relatively young SLE subjects was described [83]. In addition, CD8^+^ CD57^+^ T cells were also associated with the development of anemia in women affected by SLE [84]. Similarly, the clinical progression of RA, in terms of cognitive functions as well as articular and extra-articular manifestations, has been related to a premature immune-senescence (i.e., lack of CD28) of CD4^+^ and CD8^+^ T cells [69,85]. Notably, senescent CD4^+^ and CD8^+^ T cells have been implicated in the pathologic bone loss observed in osteolytic diseases, such as RA, juvenile idiopathic arthritis and Ankylosing Spondylitis (AS) [86,87]. According to these observations, increased levels of circulating cytotoxic CD28^−^ CD8^+^ T cells were found in patients with AS and Behçet’s disease (BD) and correlated to the disease status rather than to the age of the subjects [88,89]. Similarly, a CD28^−^ CD8^+^ T subset producing large amounts of IFN-γ was found more frequently in patients affected by Graves’ disease (GD) compared to age-matched healthy donors [90]. Moreover, signs of premature CD8^+^ T cells senescence have been reported in young patients with Primary Progressive Multiple Sclerosis (PPMS) rather than Relapsing-Remitting Multiple Sclerosis (RRMS) [91].

Taken together, these data strongly support a correlation between the premature T cell senescence and the progression of several autoimmune diseases. However, the mechanisms underlying this connection are still undefined and their elucidation could be pivotal for the development of specific therapies.

## 4. CD8^+^ T Cell Senescence and Cancer

Several works support a strict correlation between the number of CD28^−^ CD8^+^ T cells or CD57^+^ CD8^+^ T cells and cancer. In fact, such T cell subsets are highly frequent in both peripheral blood and tumor microenvironment (TME) of patients with solid tumors [92,93,94,95] and haemato-oncological malignancies [96,97]. Accordingly, CD8^+^ T cells of subjects affected by lung cancer quickly reach their senescent state by accumulating in the peripheral blood as CD28^−^ CD57^+^ T cells [98]. Moreover, chemotherapy-induced DNA damage further promotes premature senescence by negatively affecting tumor progression [99]. Likewise, in Acute Myeloid Leukemia (AML) patients, the gene expression profile of peripheral CD8^+^ T cells showed a premature senescent phenotype, characterized by the expression of CD57, and an exhausted state, supported by the upregulation of PD-1 and CTLA-4, which was remarkably enhanced after chemotherapy [100]. Notably, an enrichment of CD28^−^ CD8^+^ T cells, besides going along with T cell immunosenescence, was also found in patients with glioblastoma (GBM) [101]. Indeed, a pro-tumoral effect of this subset is suggested by the worse prognosis observed in elderly GBM patients [102,103]. Albeit GBM is known to promote the exhaustion of T cells, the failure of immune checkpoint inhibitors (ICI) treatment suggests that this CD8^+^ T cell subset is mainly senescent rather than exhausted. In fact, these cells lack the main ICI targets, PD-1 and T-cell Immunoglobulin Mucin (TIM)-3, thus explaining the failure of ICI treatment. Moreover, all senescent features appeared exacerbated in GBM by comparison to sex- and age-matched healthy subjects [101].

In patients with advanced non-small cell lung cancer (NSCLC), a high number of peripheral blood circulating CD28^−^ CD57^+^ KLRG-1^+^ CD8^+^ T cells is associated with a poor clinical response to PD-1 inhibitors [104]. This finding allows to speculate that senescent CD8^+^ T cells are involved in the resistance to ICI in these patients. Nevertheless, whether T cell immune-senescence is the cause or the effect of this resistance still remains unclear.

However, given the heterogeneity of CD28^−^ CD8^+^ T cells (i.e., production of cytotoxic or immunosuppressive factors), which depends on both the cancer type and the individual genetic background, their contribution to the tumor progression and the impact on a specific immune-strategy are unpredictable [14]. For example, immunosuppressive CD28^−^ CD8^+^ T cells expressing Forkhead box p (Foxp) 3 have been found increased in patients with non-small cell lung cancer and their frequency declines after tumor removal [105]. This CD28^−^ CD8^+^ Foxp3^+^ subset, found in many cancers, has also been identified in the TME of GBM patients, and it amplifies the immunosuppression mediated by T regulatory (reg) cells, thus contributing to the dysfunction of dendritic cells (DCs) [103,106,107,108]. On the opposite, in melanoma patients, CD28^−^ CD8^+^ T cells expressing NK associated receptors (NKRs), are able to produce perforin, thus promoting an efficient anti-tumor response [109].

Multiple Myeloma (MM) mainly affects elderly people. The malignant cells, once established in the bone marrow (BM), are sustained by several pro-inflammatory cytokines belonging to SASP [110]. In particular, IL-6 seems to favor tumor progression by preventing apoptosis of cancer cells [111]. Moreover, the tumor itself creates a pro-inflammatory microenvironment that contributes to the immune stress, thus resulting in a significant boost of senescent and exhausted T cells, especially at the tumor site. Accordingly, these senescent CD8^+^ T cells show lower proliferation and impaired functions, such as lack of IFN-γ production and degranulation upon in vitro CD3/CD28 stimulation [112]. Therefore, this pro-inflammatory TME represents a huge limit for novel immunotherapies, such as that based on Chimeric Antigen Receptor (CAR)-T cells, since once infiltrating the tumor site, these cells could lose their anti-tumor efficacy acquiring a senescent and/or exhausted phenotype [113,114,115].

Given the manifold implications of the senescent CD8^+^ T cells in the tumor context, they should be taken into account when designing anti-tumor immune-based strategies. Indeed, to potentiate anticancer therapies, it might be helpful to revert the senescence of CD8^+^ T lymphocytes through different strategies such as: (i) selective removal of senescent T cells in favor of an expansion of effector cells; (ii) reprogramming senescent T lymphocytes to make them more functional; (iii) adoptive transfer of competent T cells in order to bypass the CD28 co-stimulation required to re-differentiate stem cells into naïve and cytotoxic lymphocytes; and (iv) restoration of naïve T lymphocyte pool through a recovery of the thymic environment [103].

**Figure 1:**
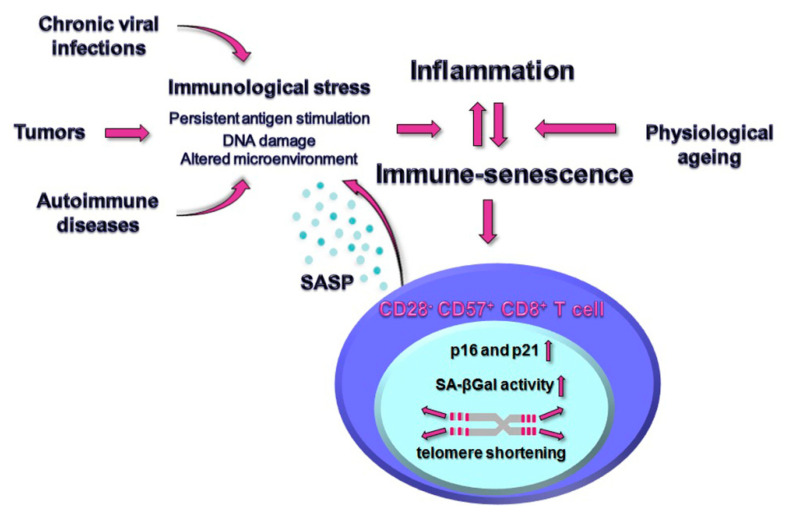
Immunological stress derived from chronic viral infections, autoimmune diseases and tumor conditions fuels the inflammation, typically of low grade during a healthy ageing, favoring the expansion of senescent CD28^−^ CD57^+^ CD8^+^ T cells that, despite the telomere shortening, the higher nuclear p16 and p21 expression, the DNA damage foci could retain proliferative capability and release proteases, interleukins, chemokines and pro-inflammatory factors that feature the specialized secretory activity termed senescence associated secretory phenotype (SASP), thus contributing to the overall inflammation. Senescence-associated β-galactosidase (SA-βGal) activity displays an age-associated increase in CD8^+^ T cell populations.

The picture emerging from the literature taken into consideration suggests that the premature T cell senescence observed during chronic viral infections, autoimmune diseases or cancer may not only be a consequence of the established inflammation, but may also exacerbate the inflammatory state, thus promoting a negative feedback loop of persistent immunological stress (Figure 1).

## 5. Conclusions

A comprehensive knowledge of CD8^+^ T cell senescence and the mechanisms underlying this process may be crucial for preventing and ameliorating several senescence-related disorders. Future work should investigate how to hamper premature and/or accelerated T senescence in order to strengthen the efficacy of several immune-based strategies as well as to guarantee a healthy ageing. Accordingly, some works have highlighted the possibility to revert such phenomenon by limiting the expansion of senescent CD8^+^ T cells. As an example, anti-TNF-α treatment could be useful to delay the loss of CD28 on CD8^+^ T cells [116]. Similarly, IL-15 was found to revert senescence in tumor antigen-specific memory CD8^+^ T cells by downregulating p16, p21 and p53 [117]. Interestingly, the combination of IL-7 and IL-15 was efficient in promoting the switch of CD8^+^ T cells towards a memory stem-like phenotype with superior survival and expansion capabilities [118]. It must be also considered, however, that CD8^+^ T cells and NK cells themselves play a role in the clearance of senescent cells. In this regard, SASP-related pro-inflammatory cytokines, especially IL-6, promote, in senescent primary human dermal fibroblasts, the upregulation of HLA-E molecules that, by interacting with the inhibitory NK group 2 member A (NKG2A) receptor expressed on NK and highly differentiated CD8^+^ T cells, allows the senescent cells to escape clearance [119]. Accordingly, the blockade of HLA-E/NKG2A interaction could be useful to prevent both the increase and the accumulation of senescent cells in inflamed tissues. In order to counteract their premature amplification and unwanted detrimental effects, a deeper profiling in healthy ageing, as well as in pathological conditions coupled with a better characterization of their behavior and crosstalk with other immune and non-immune cells, will be useful to draw more appropriate therapeutic strategies.

## 6. Search Strategy

The electronic databases of PubMed and Web of science were searched between 2014 and February 2022 (last date searched). Subject headings and keywords such as “CD8^+^ T cell”, “immunosenescence”, “inflammaging”, “infectious diseases”, “autoimmune disease”, “cancer”, and specific viruses such as “HIV”, “CMV”, “Sars-CoV-2” and specific markers such as “CD28”, “CD57”, were combined in the search.
